# Threading the barrel of the RNA exosome

**DOI:** 10.1016/j.tibs.2013.06.013

**Published:** 2013-10

**Authors:** Claudia Schneider, David Tollervey

**Affiliations:** 1 Institute for Cell and Molecular Biosciences (ICaMB), Newcastle University, Newcastle upon Tyne, UK; 2 Wellcome Trust Centre for Cell Biology, The University of Edinburgh, Edinburgh, UK

**Keywords:** exosome, RNA surveillance, RNA processing, RNA quality control, exosome cofactor

## Abstract

In eukaryotes, the exosome complex degrades RNA backbones and plays key roles in RNA processing and surveillance. It was predicted that RNA substrates are threaded through a central channel. This pathway is conserved between eukaryotic and archaeal complexes, even though nuclease activity was lost from the nine-subunit eukaryotic core (EXO-9) and transferred to associated proteins. The exosome cooperates with nuclear and cytoplasmic cofactors, including RNA helicases Mtr4 and Ski2, respectively. Structures of an RNA-bound exosome and both helicases revealed how substrates are channeled through EXO-9 to the associated nuclease Rrp44. Recent high-throughput analyses provided fresh insights relating exosome structure to its diverse *in vivo* functions. They also revealed surprisingly high degradation rates for newly synthesized RNAs, particularly RNA polymerase III transcripts.

## Highlights

• A wide range of *in vivo* targets for the exosome complex has been established.
• RNA polymerase III transcripts have emerged as major substrates.
• The human nucleus has spatially localized forms of the exosome, with matching cofactors.
• Structural analyses reveal a highly conserved RNA path through the eukaryotic exosome.

## The exosome: a versatile player in RNA maturation and decay

Almost the entire eukaryotic genome generates RNA transcripts, which participate in all aspects of gene expression and must be maintained at the correct level for any specific cellular state 1, 2. Most RNA molecules require multiple co-transcriptional and post-transcriptional processing steps, which are coordinated with the assembly and reorganization of functional RNA–protein complexes. These features mean that quality control of the fidelity of RNA processing and RNA–protein complex assembly is a substantial challenge. To meet this challenge, cells have developed a sophisticated network of RNA exonucleases and endonucleases (see Glossary) with both generic and specialized functions 3, 4. Acting together with multiple cofactors, these nucleases are capable of RNA target recognition, processing, quality control, and degradation.

In eukaryotes and Archaea, a multi-protein complex called the exosome provides the major 3′ to 5′ exonuclease activity for a wide range of substrates drawn from all nuclear and cytoplasmic 3′ to 5′ RNA degradation and processing pathways. Known roles for the eukaryotic exosome include accurate 3′ end processing of highly expressed, stable functional RNAs such as ribosomal RNAs (rRNAs) and small nuclear and nucleolar RNAs [sn(o)RNAs], turnover of diverse non-coding RNAs (ncRNAs) in the nucleus and protein-coding mRNAs in the cytoplasm, and the surveillance and degradation of aberrant RNAs of many types in both the nucleus and cytoplasm 1, 5.

Here we review recent structural and biochemical studies on the eukaryotic exosome and selected cofactors that have generated detailed information about their interplay and mechanistic properties *in vitro*. In addition, we discuss current knowledge about the target range and substrate acquisition mechanisms of the yeast exosome *in vivo*.

## The exosome: a surprising variety of enzymatic activities and cofactors

The exosome complex is an important, evolutionarily conserved factor in RNA processing and surveillance. Exosome complexes from eukaryotes and Archaea have very similar barrel-like core structures that are related to the phosphorolytic RNase complex PNPase from Eubacteria. In each case the central channel is wide enough (8–10 Å) to accommodate single-stranded RNA, but not double-stranded RNA. This suggests that RNA substrates are unwound and then threaded through the complex towards (an) internal catalytic site(s) 6, 7. This model was confirmed for the archaeal exosome core structure, in which phosphorolytic active sites are positioned on the inside faces of each of the three identical heterodimers (aRrp41 and aRrp42) that form the barrel, and RNA is threaded through this central barrel to reach the active sites in aRrp41 [7]. The barrel of the eukaryotic exosome is made up of six proteins with homology to phosphorolytic nucleases, formed into dimers resembling the archaeal Rrp41–Rrp42 pairs. It is therefore surprising that the eukaryotic exosome core is catalytically inert in most organisms tested 6, 8, 9. This finding suggests that the eukaryotic exosome core might merely form a scaffold for the association of active nucleases and numerous cofactors, which assist the exosome in target recognition, unwinding, and degradation. However, this interpretation is difficult to reconcile with the observation that the core exosome subunits are all strictly essential for viability in yeast, and show high evolutionary conservation on both the outside and inside of the barrel structure. The relationship between the eukaryotic exosome core structure and the catalytic activity of the holoenzyme *in vivo* is therefore unclear.

### Composition of the eukaryotic exosome

**Figure 1:**
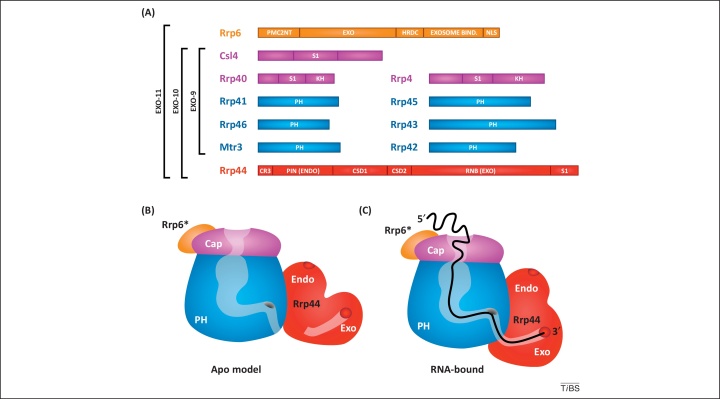
The RNA pathway through the eukaryotic exosome. **(A)** Domain structures of budding yeast exosome components and composition of exosome subcomplexes EXO-9, EXO-10, and EXO-11. The following domains are indicated: EXO, domain harboring exonuclease activity, homologous to RNase D (in Rrp6) or RNase II/R (in Rrp44, RNB domain) from Eubacteria; ENDO, domain harboring endonuclease activity in Rrp44 (PIN, PilT N terminus); HRDC, helicase and RNase D C-terminal domain, putative role in RNA-binding; Exosome Bind, region in Rrp6 that interacts with EXO-10; PMC2NT, domain found at the N terminus of 3′ to 5′ exonucleases with HRDC domains, also called the NUC016 domain; NLS, nuclear localization signal; S1, RNA-binding domain, originally identified in the ribosomal RNA-binding protein S1; KH, RNA-binding domain with K-homology, originally identified in the RNA-binding protein hnRNP K; PH, domain with homology to RNase PH from Eubacteria; CR3, protein motif containing three Cys residues; CSD, cold-shock RNA-binding domain. Proteins forming the cap and hexameric ring of EXO-9 (see panels B and C) are shown in purple and blue, respectively. Associated nucleases are depicted in red (Rrp44) or orange (Rrp6). **(B)** Illustration of the apo (RNA-free) form of the yeast exosome. The model is inferred from the structure of the Rrp44–Rrp41–Rrp45 trimeric complex and contains a C-terminal fragment (Exosome Bind) of Rrp6 (Rrp6*) 12, 77. The cavity within the exosome barrel is indicated in a lighter shade of purple or blue, respectively, and this channel is not predicted to be connected to the exonuclease active site of Rrp44 in the RNA-free apo form. The darker gray lozenge indicates the position in yeast Rrp41 that corresponds to the active site of archaeal Rrp41. **(C)** Schematic of RNA threading through the exosome channel to the exonuclease active site of Rrp44 via the inactive catalytic center of yeast Rrp41 (darker gray lozenge, see panel B). The cartoon is based on the 2.8-Å resolution structure of a catalytically inactive yeast EXO-11 complex bound to an RNA [77] (image modified from a generous personal communication from E. Conti). The RNA is shown in black, and structured regions near the 5′ end of the substrate are portrayed as being unwound by the cap proteins. The exonuclease domain of Rrp44 adopts a closed conformation upon RNA binding that captures the 3′ end of the RNA in close vicinity to the active site.

In contrast to the core phosphorolytic RNase activity of the archaeal exosome and the bacterial PNPase, the nuclease activity of eukaryotic complexes is hydrolytic and dependent on the association of enzymatic subunits with the core structure. Early structural studies on budding yeast and human exosome complexes revealed a two-layered, barrel-like core composed of nine catalytically inert subunits (EXO-9, Figure 1) [6], although EXO-9 can be catalytically active in some plants [10]. The upper layer of EXO-9 is composed of a cap of three proteins (Csl4, Rrp4, and Rrp40 in yeast) with S1 and/or KH RNA-binding domains. The cap rests on a ring of six proteins that have homology to RNase PH from Eubacteria (Rrp41, Rrp45, Rrp46, Rrp43, Mtr3, and Rrp42 in yeast) (Figure 1). The active nuclease Rrp44 (also known as Dis3) is homologous to bacterial RNase II/R and is located on the base of the core barrel 11, 12, forming the EXO-10 complex. In yeast, EXO-10 is present throughout the cell, whereas a nuclear-restricted complex (EXO-11) contains an additional nuclease Rrp6, with homology to bacterial RNase D.

### An unexpected endonuclease activity in the exosome

When the yeast exosome was identified more than 15 years ago [13], its name was derived from the observation that the complex exhibited 3′ to 5′ exonuclease activity both *in vitro* and *in vivo*. Exonuclease active sites were localized to both Rrp44 and Rrp6, but subsequent studies identified an additional endonuclease activity within Rrp44. The 3′ to 5′ exonuclease activity of Rrp44 resides in an RNB domain (named after the gene locus encoding *Escherichia coli* RNase II), which is located towards the C terminus (Figure 1A). By contrast, the N-terminal PIN (PilT N terminus) domain harbors endonuclease activity and functions in tethering Rrp44 to the EXO-9 core, at least in yeast and fruit flies 11, 14, 15, 16, 17. PIN domains have been characterized in several RNA processing and surveillance factors, where they show metal-dependent endonuclease activities [4]. The endonuclease activity of Rrp44 requires a non-physiological concentration (5 mM) of manganese ions *in vitro*. This is also the case for other eukaryotic PIN domain nucleases tested. In addition, the very N terminus of Rrp44 consists of a CR3 motif (a small domain with three conserved Cys residues) that, together with the PIN domain, mediates the interaction between Rrp44 and EXO-9 in yeast and modulates its enzymatic activities 16, 18.

### Diverse catalytic subunits in plants and vertebrates

There is only one copy each of Rrp6 and Rrp44 in fungi and invertebrates, but three RRP6-like proteins have been identified in *Arabidopsis thaliana*. Two of the plant RRP6 variants exhibit specialized intranuclear localizations and functions, whereas the third variant is restricted to the cytoplasm [19]. Similarly, human cells express three different RRP44/DIS3 isoforms that have distinct subcellular locations and activities. hDIS3 is most closely related to yeast Rrp44 in sequence, and exhibits both exonuclease and endonuclease activity. The localization of hDis3 is mainly nuclear, with nucleolar exclusion, whereas DIS3L1 (DIS3-like protein 1, also known as DIS3L) and DIS3L2 are strictly cytoplasmic and show exonuclease but not endonuclease activity 20, 21, 22, 23, 24. DIS3 and DIS3L1 both associate with EXO-9 20, 21, in contrast to DIS3L2, which functions independently of the exosome in both humans and fission yeast 22, 23, 24, 25.

The apparent lack of any DIS3 isoform in the human nucleolus, the key location for early steps of ribosome biogenesis, was unexpected given that yeast Rrp44 and Rrp6 have prominent, non-redundant roles in pre-rRNA processing. hRRP6 is found throughout the nucleus and, to a small extent, in the cytoplasm, but is enriched in the nucleolus. The apparent nucleolar exclusion of hDIS3 suggests that nucleolar pre-rRNA processing steps are performed only by RRP6 in humans. Moreover, it was recently shown that hRRP6 plays a key role in removal of the structured, pre-ribosomal internal transcribed spacer 1 (ITS1) region from the 3′ end of 18S rRNA. This function is not conserved in yeast, in which the equivalent steps are endonucleolytic, indicating that hRRP6 has acquired additional roles in pre-rRNA processing 26, 27. Notably, hRRP6 is better able to productively engage and degrade structured substrate than yRrp6 is. Moreover, hRRP6 activity is probably modulated by post-translational phosphorylation, not present in yeast, which can regulate release of the active site from a non-productive or dormant state [28].

Consistent with their nuclear/cytoplasmic distributions, hDIS3 and hRRP6 participate in the nuclear degradation of unstable, promoter-associated transcripts (PROMPTs), whereas DIS3L1 degrades cytoplasmic rRNA that has undergone polyadenylation 20, 21, 29. Notably, the non-exosome-associated DIS3L2 protein degrades a distinct set of substrates in the cytoplasm, including mRNAs and miRNA precursors, and has a preference for 3′ uridylated RNA substrates both in humans and in fission yeast 23, 24, 25.

### Subcellular partitioning of exosome cofactors

**Figure 2:**
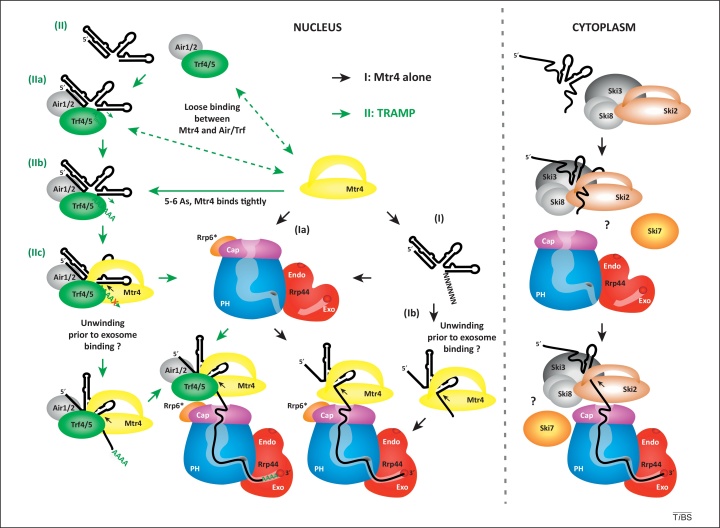
Model of interactions between the exosome and helicase-containing cofactors: threading in tandem? Nucleus (left): In preparation for degradation by the nuclear exosome, structured substrates are unwound by the DExH-box helicase Mtr4 and threaded into the exosome central channel. Route I (Mtr4 alone, black arrows): If the RNA substrate contains a sufficiently long 3′ overhang of approximately 5–6 nt **(I)**, Mtr4 can bind and act on its own. It is currently unclear whether Mtr4 associates with the exosome prior to RNA binding **(Ia)** or whether unwinding by Mtr4 requires the presence of the exosome *in vivo***(Ib)**. Route II (TRAMP complex, green arrows): Substrates with shorter 3′ extensions **(II)** are targets for the TRAMP complex and are bound by Air1 or Air2 and oligoadenylated by the poly(A) polymerase Trf4 or Trf5 **(IIa)** until a minimal binding site for Mtr4 is generated **(IIb)**. Mtr4 is believed to loosely associate with the remaining components of the TRAMP complex on structured substrates. This association becomes more stable when the oligo(A) tails added by Trf4 or Trf5 reach the optimum of 5–6 nt, at which point Mtr4 binding inhibits further oligoadenylation **(IIc)**. Mtr4 functions better on short 3′ extensions, so TRAMP favors the production of substrates that are optimal for unwinding. Consistent with this functional interplay between polymerase and helicase activities, *in vivo* crosslinking of RNA to Trf4 revealed the predominance of very short oligo(A) tails (A_1–5_) on TRAMP substrates. Cytoplasm (right): Analogous to the Trf4/5–Air1/2–Mtr4 polyadenylation (TRAMP) complex in the nucleus, the DExH box helicase Ski2 in the cytoplasmic Ski2/3/8 complex is believed to prepare exosome substrates for threading. An additional factor, Ski7, is related to translation-associated GTPases and may aid in recruitment of Ski2/3/8 and the exosome to ribosomes. DExH, subgroup of helicase protein family; Ski, superkiller; A, adenosine; Rrp6*, exosome binding fragment of Rrp6.

In budding yeast, two related DExH-box RNA helicases, Mtr4 and Ski2, are important activators of the exosome in the nucleus and cytoplasm, respectively. Mtr4 can function alone to assist substrate unwinding in the nucleus, but it also acts as a component of the Trf4/5–Air1/2–Mtr4 polyadenylation (TRAMP) complexes (Figure 2). In addition to Mtr4, TRAMP complexes contain a poly(A) polymerase, either Trf4 or Trf5, and a Zn-knuckle RNA-binding protein, either Air1 or Air2 30, 31, 32, 33, 34, 35, 36, 37, 38. In the yeast cytoplasm, the DExH-box helicase Ski2 associates with two putative RNA-binding proteins to form the heterotetrameric SKI complex, which contains one copy each of Ski2 and the large tetratricopeptide (TPR) protein Ski3, and two copies of the small WD repeat protein Ski8 [39]. The SKI complex is strictly cytoplasmic in yeast and is required for both mRNA 3′ turnover and mRNA surveillance by the EXO-10 complex 1, 3, 5; and it also participates in RNA interference in metazoans [40]. In addition, Ski8 homologs play SKI-complex-independent roles in the nucleus of some species that are linked to meiotic recombination (in yeast) or nuclear mRNA turnover (in plants) [41].

Recent crystal structures of yeast Mtr4 and Ski2 have revealed the presence of large, β-barrel insertion domains in both helicases 42, 43, 44. These insertions are located at similar positions in the core helicase structures of Mtr4 and Ski2, and are important for interactions with substrate RNAs. However, they show significant differences in structure and RNA-binding preferences, suggesting that they have evolved distinct properties tailored to the specific targets of the nuclear and cytoplasmic exosome (Figure 2) 36, 42, 43, 44, 45.

In the human nucleus, exosome cofactors show localization to specific subnuclear regions that appear to correlate with the localization of exosome-associated nucleases (Table 1). The putative homologs of the yeast TRAMP subunits are hTRF4-2 (Trf4), ZCCHC7 (Air2), and hMTR4 (Mtr4) 46, 47, 48 and the function of hAIR2/ZCCHC7, at least, appears to be restricted to the nucleolus. hMTR4 is also present in another trimeric nuclear complex (nuclear exosome targeting; NEXT), together with a different Zn-knuckle protein, ZCCHC8, and the putative RNA-binding protein RBM7. Both ZCCHC8 and RBM7 are excluded from nucleoli, and NEXT functions in the nucleoplasm to promote degradation of PROMPTs by the exosome [46]. This suggests that the human TRAMP and NEXT complexes function specifically in the nucleolus and nucleoplasm, respectively. Notably, hMTR4 can also associate with three other Zn-knuckle proteins, ZFC3H1, ZC3H18, and ARS2, suggesting that multiple related nuclear complexes may act as exosome cofactors in human cells [46].

**Table 1:** Comparison of exosome components and cofactors in yeast and human cells

	Nucleolus		Nucleoplasm		Cytoplasm	
	Yeast	Human	Yeast	Human	Yeast	Human
**Nuclease**						
**EXO (RNase II/R)**	Rrp44 (Dis3)	–	Rrp44 (Dis3)	DIS3	Rrp44 (Dis3)	DIS3/DIS3L1 DIS3L2
**ENDO (PIN, inactive)**	Rrp44 (Dis3)	–	Rrp44 (Dis3)	DIS3	Rrp44 (Dis3)	DIS3/DIS3L1 DIS3L2
**EXO (RNase D)**	Rrp6	hRRP6	Rrp6	hRRP6	–	hRRP6
**Poly(A) polymerase**						
**Non-canonical**	Trf4 (Pap2) [TRAMP4]	hTRF4-2 [TRAMP]	Trf4 (Pap2) [TRAMP4]	–	–	–
**Non-canonical**	Trf5 [TRAMP5]	hTRF4-1 [?]	Trf5 [TRAMP5]	hTRF4-1 [?]	–	–
**RNA helicase**						
**DExH**	Mtr4 [TRAMP]	hMTR4 (SKIV2L2) [TRAMP]	Mtr4 [TRAMP]	hMTR4 (SKIV2L2) [NEXT] hSKI2 (SKIV2L) [SKI]	Ski2 [SKI]	hSKI2 (SKIV2L) [SKI]
**Other**	Sen1 [Nrd1–Nab3–Sen1]	Senataxin [?]	Sen1 [Nrd1–Nab3–Sen1]	Senataxin [?]	–	Senataxin [?]
**RNA-binding**						
**Zn-knuckle**	Air1 [TRAMP] Air2 [TRAMP]	ZCCHC7 [TRAMP] ZFC3H1 [?] ZC3H18 [?] ARS2 [?]	Air1 [TRAMP] Air2 [TRAMP]	ZCCHC8 [NEXT] ZFC3H1 [?] ZC3H18 [?] ARS2 [?]	–	–
**Sas10_Utp3**	Rrp47 (Lrp1)	C1D	Rrp47 (Lrp1)	C1D	–	–
**Other**	–	–	–	RBM7 [NEXT]	–	–
**Other**	Mpp6	hMPP6	Mpp6	hMPP6	–	–
**RRM**	Nrd1 [Nrd1–Nab3–Sen1]	–	Nrd1 [Nrd1–Nab3–Sen1]	–	–	–
**RRM**	Nab3 [Nrd1–Nab3–Sen1]	–	Nab3 [Nrd1–Nab3–Sen1]	–	–	–
**Other**						
**TPR**	–	–	–	hSKI3 [SKI]	Ski3 [SKI]	hSKI3 [SKI]
**GTPase**	–	HBS1L [?]	–	HBS1L [?]	Ski7	HBS1L [?]
**WD-repeat**	Ski8 [?]	hSKI8 (WDR61) [?]	Ski8 [?]	hSKI8 (WDR61) [SKI]	Ski8 [SKI]	hSKI8 (WDR61) [SKI]

Alternative names are given in brackets. Complexes are indicated in square brackets, where known.

Human homologs exist for the yeast Ski2/3/8 complex components and the hSKI complex localizes to both cytoplasm and nucleus [49]. In yeast, the Ski2/3/8 complex interacts with Ski7, which is required for its function in mRNA turnover and surveillance 1, 3, 5. Ski7 resembles translation-associated GTPases, eukaryotic elongation factor 1α (eEF1α) and eukaryotic release factor 3 (eRF3), suggesting that it mediates interactions between the Ski2/3/8 exosome complex and mRNA-associated ribosomes. Ski7 homologs have not been identified in many fungi and metazoans, where Ski7 may be replaced by the related GTPase Hbs1 (HBS1L in humans) [21].

### Links between the exosome, TRAMP, and the 3′-end processing machinery

In addition to their roles in surveillance and degradation, the exosome and TRAMP complexes function in specific RNA maturation steps. In yeast, these include 3′ end formation on snoRNAs and on some mRNAs (e.g., *NAB2* and *CTH2*) that are processed from 3′ extended precursors. This may ensure that incorrect transcripts are rapidly eliminated, and competition between surveillance involving Rrp6 and polyadenylation can regulate the expression of these genes 50, 51, 52, 53, 54, 55, 56, 57. This decision can be modulated by the concentration of the nuclear poly(A) binding protein Nab2; elevated levels of Nab2 promote degradation of *NAB2* mRNA by Rrp6 [53], with which it physically interacts [58]. Conversely, Rrp6 can displace Nab2 from nuclear poly(A) tails, suggesting that nuclear mRNP biogenesis is monitored by the exosome, influencing both the polyadenylation efficiency and association with poly(A) binding proteins [58].

Taken together, recent research has uncovered an unexpected endonuclease activity in the eukaryotic exosome, and has highlighted a surprisingly high level of specialization for the two catalytic exosome subunits and their cofactors in different subcellular compartments, especially in multicellular organisms. Furthermore, it is now clear that the ‘conventional’ 3′-end processing machineries are functionally intertwined with RNA surveillance factors.

## Three catalytic activities in one complex: who does what and how?

Numerous *in vitro* analyses of the yeast exosome have compared individual subunits to the reconstituted complexes 6, 8, 9, 11, 14, 15, 16, 18, 59, 60, 61, 62, 63, 64. These have generally been aimed at defining the molecular mechanisms by which substrates are recognized and degraded by the exonuclease activities of Rrp6 and Rrp44 and the endonuclease activity of Rrp44 within the exosome complex. Particularly intriguing is the role of Rrp6, which acts within the context of EXO-11 but also has exosome-independent targets, because many known functions of Rrp6 are not impaired by loss of any EXO-10 component 64, 65, 66, 67, 68, 69. Differential requirements for the endonuclease and exonuclease activities of Rrp44 have been reported for small numbers of specific substrates, for example in the degradation of normal versus non-stop mRNAs [60]. However, in most cases the individual substrates targeted to each of the three catalytic activities in the exosome and their routing to the appropriate catalytic site remain unclear.

### Novel transcriptome-wide approaches define the target range of the yeast exosome

Initial analyses of exosome targets were generally limited to subsets of known substrates. However, comprehensive views of exosome targets have been provided by a combination of two more recent techniques. High-resolution tiling arrays have been used to determine changes in the yeast transcriptome on loss of Rrp6 65, 70, 71 or in the presence of different combinations of catalytic mutations in Rrp6 and Rrp44/Dis3 [72]. These analyses identified a plethora of novel exosome substrates, and large numbers of stable un-annotated and cryptic unstable transcripts (SUTs and CUTs, respectively) in particular. The tiling arrays revealed that Rrp44 and Rrp6 have both overlapping and specific roles in degrading distinct classes of substrates. CUTs and SUTs, for example, are targeted to both Rrp44 and Rrp6, whereas the two nucleases appear to have distinct roles in the processing of sn(o)RNAs. Unexpectedly, the microarray analyses also indicated that the catalytic activities of Rrp44 are required to degrade large amounts of intron-containing pre-mRNAs and tRNA precursors, even in the absence of evident processing defects [72]. In *Drosophila*, microarray analyses showed that different mRNAs can be preferentially sensitive to loss of Rrp6, Rrp44/Dis3, or core exosome subunits [73].

The second approach was the application of *in vivo* RNA crosslinking and analysis of cDNAs (CRAC) to nuclease components of the yeast exosome (Rrp44 and Rrp6), two structural subunits (Rrp41 and Csl4), and a cofactor (Trf4) 69, 74. Crosslinking of wild type Rrp44 and catalytic mutants identified many classes of substrates that are targeted to the exonuclease activity of Rrp44, including the CUT and SUT classes of ncRNA, snoRNAs, and, most prominently, pre-tRNAs and other Pol III transcripts. Pol III RNAs also emerged as major targets in crosslinking analyses of the exosome cofactor complex Nrd1–Nab3 74, 75. Both microarray and crosslinking analyses therefore indicate that wild type cells discard a substantial fraction of newly synthesized RNAs via Rrp44-dependent exosome degradation, particularly RNAs transcribed by RNA Pol III. This indicates that the kinetic proofreading pathway that helps to ensure the efficiency and accuracy of RNA processing is even more active than anticipated.

### RNA threading is conserved between eukaryotic and archaeal exosome structures

Structural analyses of exosome subunits or subcomplexes, as well as *in vitro* RNase protection experiments, had previously suggested that substrates can reach the Rrp44 exonucleolytic active site following passage through the lumen of the exosome barrel structure, analogous to the RNA path described for the archaeal exosome 7, 12, 76. The 2.8-Å resolution structure of a catalytically inactive yeast EXO-11 complex bound to RNA has recently been reported [77]. This confirmed that a model RNA could indeed be funneled into the EXO-9 channel in a single-stranded conformation, which is promoted by an unwinding pore formed by the cap proteins (Figure 1C). Inside the channel, the RNA forms a series of sequence-independent interactions, similar to contacts seen in the archaeal exosome and consistent with the heterogeneous nature of exosome substrates. Surprisingly, the pathway followed by RNA through the barrel brings it into close proximity with the region of Rrp41 that forms the active site in the archaeal core exosome, which is catalytically inactive in yeast. Consistent with this finding, yRrp41 is notably better conserved to archaeal Rrp41 and the bacterial phosphorolytic nuclease RNase PH than are the other core exosome subunits.

Why these structural features of Rrp41 should have been so strikingly conserved in the absence of a conserved nuclease function was a mystery, which has now been partly resolved by recent structural data. At the exit site from the central barrel of EXO-9, the exonuclease domain of Rrp44 adopts a closed conformation on RNA binding that captures the 3′ end of the RNA in close vicinity to the active site (compare Figure 1B and 1C). Threading appears to shield the RNA from the endonuclease active site of Rrp44, which faces the solvent 12, 77. Despite this, it was reported that the endonuclease activity releases natural exosome substrates that are probably threaded through the barrel and stalled at sites of strong secondary structure 14, 69. Moreover, *in vivo* crosslinking performed using cleavable Rrp44 proteins (split-CRAC) suggested that Rrp44 endonuclease and exonuclease activities cooperate on most substrates [69]. However, some elements of the Rrp44 PIN domain that are outside the endonuclease site contribute to the long RNA path through the central channel seen in the crystal structure, and this may be responsible for crosslinking to many threaded substrates [77]. No alternative path connecting the two active sites in Rrp44 has been proposed, but it is notable that the current structure contains a mutant form of Rrp44 that is devoid of both catalytic activities [77], and structural rearrangements may occur in the active EXO-11 complex. In the RNA-free crystal structure of Rrp44–Rrp41–Rrp45, the relative position of Rrp44 is significantly different from that in the RNA-bound form (Figure 1B). Further changes may be anticipated when the highly processive exonuclease domain of Rrp44, analogous to its bacterial homologs RNase II/R, actively pulls the substrate RNA though the complex [78].

The overall importance of RNA threading through the channel is supported by the finding that mutations occluding the channel negatively affect all three exosome RNase activities in the context of EXO-10 and EXO-11 *in vitro*, and this has been confirmed *in vivo* for selected exosome substrates 12, 59, 64. It is, however, not known what fraction of natural substrates follow the threading path or how the endonuclease activity of Rrp44 is affected by channel occlusion.

Taken together, these results suggest that although the chemistry of ribonucleolytic activities involved is different, the channeling mechanism for eukaryotic exosome complexes appears to be remarkably well preserved with respect to both the archaeal exosome and bacterial PNPase. Moreover, all three machineries mirror fundamental mechanisms used by the proteasome to prepare polypeptides for processive degradation [77].

### Alternative entry sites for structured and unstructured substrates?

The crystal structure of yeast EXO-11 bound to RNA includes only a small C-terminal portion of the nuclear exonuclease Rrp6 [77]. Consistent with previous studies [79], this is located on the ‘top’ of the exosome, distant from Rrp44 and close to the cap protein Csl4 (Figure 1). Rrp6 activity appears to depend on the upper portion of the channel and it has been hypothesized that RNA passes through the S1/KH ring to access the catalytic site of Rrp6 [59]. Without making direct contact to the RNA in the EXO-11 crystal structure, the C-terminal region of Rrp6 is predicted to extend the RNA path through the channel and stabilize binding of the substrate to the exosome core [77]. Supporting this model, both RNase activities of Rrp44 were stimulated by the presence of Rrp6 *in vitro*, and Rrp6 itself is also inhibited by a mutation in the Rrp44 exoribonuclease active site in EXO-11 [59]. Although these *in vitro* studies on model RNAs imply cooperation between all three catalytic activities in the nuclear exosome on threaded substrates, the number of *in vivo* targets affected by this is currently unclear [59].

*In vivo* crosslinking revealed that structured RNA targets are preferentially associated with Rrp6 [69], which is located close to the exosome cap proteins that are important for unwinding of a threaded model substrate [77]. However, the model in which substrates destined for Rrp6 initially enter the central channel, but then exit via a side pore to reach the active site of Rrp6 [59], seems difficult to reconcile with the current EXO-11 crystal structure [77]. Moreover, *in vivo* crosslinking studies revealed that highly structured Rrp44-dependent RNA substrates are often associated with both Rrp6 and Rrp44, with little or no contact to the remaining core exosome. This suggests the use of an alternative entry site to the Rrp44 catalytic center for some substrates, possibly aided by docking to Rrp6 and other exosome cofactors [69]. Such an alternative entry site can be fitted onto the Rrp44–Rrp41–Rrp45 crystal structure [12]. The basis for the distinction between threaded and docked substrates is unclear, but a long (∼33 nt) single-stranded region is required to access the exonuclease domain of Rrp44 via threading through the barrel, whereas much shorter single-stranded regions would be sufficient for direct access to the catalytic sites of Rrp44 or Rrp6.

### Threading in tandem?

There are many reported links between Rrp6 and the TRAMP complex, which helps to unwind nuclear structured substrates 30, 45, and it has been proposed that the Mtr4 helicase in the TRAMP complex is located on top of the exosome, close to Rrp6 77, 79 and the RNA entry site 42, 43. In both the nucleus and cytoplasm, the functions of the core EXO-10 complex appear to be largely or entirely dependent on the DExH box helicases Mtr4 (in the nucleus) and Ski2 (in the cytoplasm). Recent structural analyses of these factors indicate that, like the exosome itself, they form a pore through which the RNA substrate is likely to pass 42, 43, 44. This suggests the possibility that the TRAMP and SKI complexes, which contain Mtr4 and Ski2, respectively, may sit on top of the exosome barrel and feed RNA directly into the exosome lumen (Figure 2). A structural rearrangement is presumably required for the 3′ end of the substrate RNA to be withdrawn from either complex prior to transfer to the exosome. The precise mechanism remains unclear, especially with respect to the SKI complex, but current data for the TRAMP complex suggest that Mtr4 remains bound to the substrate but dissociates from Air–Trf prior to binding to the exosome. This would imply that TRAMP is assembled and disassembled in each round (Figure 2). Consistent with this model, Trf4/5 show stable association with Air1/2, whereas the association of these complexes with Mtr4 is more labile. This model also provides a potential explanation for the long-standing observation that mutation of Mtr4 impairs many exosome functions that are not clearly affected by loss of Air or Trf proteins, showing that Mtr4 has both TRAMP-dependent and -independent functions. The Arch/KOW domain of Mtr4 is important for exosome activation by TRAMP, suggesting direct interactions during degradation.

RNA can be threaded through the narrow pore into the exosome core without cofactors *in vitro*, but it seems likely that ATP hydrolysis by RNA helicases greatly accelerates this process *in vivo*. It is perhaps notable that the widely conserved Ro protein has an apparently related role in threading RNA substrates through a central cavity [80], suggesting that this may be a common theme in RNA quality control.

In summary, the recent crystal structure of an RNA-bound yeast exosome and some of its cofactors, together with numerous in-depth *in vitro* analyses of these complexes, has provided invaluable insights into the mechanism by which active nucleases reach their target RNAs. In parallel, novel high-throughput approaches have been applied to systematically identify the complete set of exosome substrates in budding yeast. However, we are only starting to comprehend which catalytic activity is responsible for which substrate.

## Concluding remarks

In the 15 years since its initial identification, many key features of exosome structure and function have been defined by biochemical analyses. We now know that the exosome harbors three distinct hydrolytic activities and that the relative importance of these varies significantly between different subcellular compartments and organisms, especially in metazoans. Exosome activity depends on cofactors, which also show specialization in localization and targeting. This organization provides the versatility needed for the exosome to cope with the huge variety of RNA substrates in the cell. The combination of structural and genome-wide analyses has further increased our understanding of targeting mechanisms and provided a comprehensive view of the exosome substrate range and specificities *in vivo*.

However, detailed *in vivo* analyses remain technically challenging and many unresolved questions remain. In particular, how and why are specific substrates targeted to each of the active sites? What is the full target range for the exosome in multicellular organisms? How does the exosome decide to either accurately process or completely trash a particular substrate? Exosome components undergo post-translational modification; does this affect activity or substrate specificity? The importance of these questions is underlined by recent studies linking exosome function to several other cellular pathways, including an unexpected role in generating antibody diversity [81]. Moreover, clinical studies reveal the importance of the exosome in disease. For example, 10% of all patients suffering from multiple myeloma carry mutations that inhibit the exonuclease activity of Rrp44 [82]. Given the apparent specialization of human exosome subunits and cofactors 20, 21, 22, 83, there seems little doubt that further functions and targets of the human exosome remain to be discovered.

## Glossary

Endonucleases and exonucleases: enzymes that degrade nucleic acids can have two fundamentally different mechanisms. Endonucleases break the RNA or DNA backbone at an internal site, releasing two fragments. Exonucleases degrade the RNA or DNA from either the 5′ end (5′ exonucleases) or 3′ end (3′ exonucleases), releasing single nucleotide products.
Hydrolytic RNases: enzymes with this activity include eukaryotic Rrp44/Dis3 and Rrp6, which are homologous to bacterial RNase II/R and RNase D, respectively. A hydroxyl group is used to attack and break the target RNA backbone, releasing nucleotide 5′ monophosphate products. This reaction is strongly favored energetically, potentially providing substantial free energy to allow processive degradation.
*In vivo* RNA-protein crosslinking: deep sequencing of cDNA libraries generated from UV-crosslinked RNA fragments allows a genome-wide analysis of the *in vivo* interactome of RNA-associated proteins. This technique has recently been applied to characterize the RNA substrate specificity and targeting mechanisms of catalytic and structural exosome subunits and several exosome cofactors.
Phosphorolytic RNases: enzymes with this activity include bacterial PNPase and RNase PH, as well as the archaeal exosome core. In the reaction mechanism, phosphate is used to attack and break the target RNA backbone, releasing nucleotide 5′ diphosphate products. This reaction is almost energetically neutral, allowing phosphorolytic enzymes to act reversibly, under some conditions, polymerizing nucleotide 5′ diphosphates onto the 3′ ends of substrate RNAs. Due to the relative nucleotide abundance *in vivo*, this activity normally generates A-rich oligonucleotide tails.
PIN (PilT N terminus) domain: this motif is named after an *Escherichia coli* protein involved in pilus formation. The PIN domain harbors metal-dependent endonuclease activity in Rrp44/Dis3 and other characterized RNA processing and surveillance factors, and involves Mn^2+^ coordinated by three or four acidic residues in the release of nucleotide 5′ monophosphate products.
Tiling array: this technique uses microarray chips that carry very large numbers of oligonucleotide probes. These can cover both strands of the entire yeast genome with overlap (tiling) between adjacent probes. Labeled RNA or cDNA is hybridized to the oligonucleotides to give quantitative genome-wide mapping information. Tiling arrays have recently been applied to define substrate specificity for the three catalytic activities in the yeast exosome.
Transcriptional noise: non-coding RNAs (ncRNAs) are generated all over the eukaryotic genome and contribute to transcriptional and post-transcriptional gene regulation. In yeast, a plethora of recently identified nuclear ncRNAs with a wide range of lengths and often unknown function include cryptic unstable transcripts (CUTs), which were originally detected in strains lacking Rrp6, and stable un-annotated transcripts (SUTs). Examples in metazoans include the unstable promoter-associated transcripts (PROMPTs).
